# Combination prediction method of students’ performance based on ant colony algorithm

**DOI:** 10.1371/journal.pone.0300010

**Published:** 2024-03-11

**Authors:** Huan Xu, Min Kim

**Affiliations:** 1 Department of Public Teaching, Hefei Preschool Education College, Hefei, China; 2 Department of Youth Education and Counseling, Soonchunhyang University, Asan-si, Choongchungnam-do, Korea; TU Wien: Technische Universitat Wien, AUSTRIA

## Abstract

Students’ performance is an important factor for the evaluation of teaching quality in colleges. The prediction and analysis of students’ performance can guide students’ learning in time. Aiming at the low accuracy problem of single model in students’ performance prediction, a combination prediction method is put forward based on ant colony algorithm. First, considering the characteristics of students’ learning behavior and the characteristics of the models, decision tree (DT), support vector regression (SVR) and BP neural network (BP) are selected to establish three prediction models. Then, an ant colony algorithm (ACO) is proposed to calculate the weight of each model of the combination prediction model. The combination prediction method was compared with the single Machine learning (ML) models and other methods in terms of accuracy and running time. The combination prediction model with mean square error (MSE) of 0.0089 has higher performance than DT with MSE of 0.0326, SVR with MSE of 0.0229 and BP with MSE of 0.0148. To investigate the efficacy of the combination prediction model, other prediction models are used for a comparative study. The combination prediction model with MSE of 0.0089 has higher performance than GS-XGBoost with MSE of 0.0131, PSO-SVR with MSE of 0.0117 and IDA-SVR with MSE of 0.0092. Meanwhile, the running speed of the combination prediction model is also faster than the above three methods.

## 1. Introduction

Students’ performance serves not merely as a crucial criterion for assessing the quality of teaching, but also as a significant indicator of their proficiency in the acquired knowledge. The examination and evaluation of students’ studies and teachers’ teaching can feed back to teaching activities and teaching strategies [1]. If teachers can detect students’ learning abnormality in advance, it is possible to reduce the consequences of abnormality through guidance and intervention. In recent years, the rapid growth of the number of students makes the problem of insufficient number of full-time teachers in colleges particularly prominent. In addition, the current open teaching management mode gives students more and more freedom in learning. Students are free to set learning plans and goals. As a result, teachers cannot grasp the learning status of each student, which affects the teaching quality and students’ performance. Therefore, it is very important to accurately predict students’ performance in education management. There are many factors that affect a students’ performance. However, at present, the processing of students’ performance in colleges generally remains in simple database management and query. The analysis of students’ performance is only carried out calculating the mean and variance. Therefore, it is urgent to study how to predict and analyze students’ performance according to their daily learning behavior.

There are many factors that affect students’ performance, including external factors such as social environment, family environment, school education, etc., and internal factors of students, such as intelligence factors, physical health, psychological factors, personality factors, knowledge background, behavior habits, etc. From the perspective of psychology, the main factor that affects the performance is the student learning behavior. Student learning behavior includes the process of learning and various activities [2]. Students’ learning process refers to all behavior forms and methods used by students in the process. It principally includes learners’ emotion, thinking content, motivation, ability and specific behavior of the program. In addition, learning behavior includes not only explicit behavior changes, but also implicit behavior changes, such as learning interests, learning strategies, learning emotions, learning confidence, etc.

Nowadays more and more researchers begin to focus on the relationship between students’ performance and learning behavior. Marbouti et al. [3] used classroom attendance and combined with tests and weekly assignments to predict whether students were at risk of failing the course. The results show that classroom attendance behavior is one of the influencing factors of course performance. Similarly, Conard [4] found that classroom attendance is one of the important factors affecting students’ performance. In addition to learning behavior, living habits also affect students’ performance to a certain extent, such as diet, internet behavior and consumption behavior. Bonnardel et al. [5] studied the relationship between behavior patterns and students’ academic performance, and revealed the correlation between the two. Anne et al. [6] excavated and analyzed the flow records of college students’ campus cards. The research found that the regularity of daily life on campus is closely related to their academic performances, and pointed out that students who eat breakfast on time are more likely to get excellent academic performances.

Prediction on student’s performance refers to the technology of predicting students’ future learning performance based on existing data. It is one of the earliest and most commonly used applications in the field of educational data mining. There are two common performance prediction methods. The first is based on probability statistical models. Elbadrawy et al. [7] applied collaborative multi-regression models for predicting students’ performance in course activities. Sravani and Bala [8] used linear regression model for the prediction on student’s performance.

The second is ML-based prediction methods, such as decision tree [9], logistic regression [10], artificial neural network [11], support vector regression [12], and so on. Ramanan et al. [13] developed a learning algorithm based on functional-gradient boosting methods for logistic regression, and the empirical evaluation on standard data sets demonstrated the superiority of the proposed approach over other methods for learning LR. Zhang et al. [14] proposed attribute and instance weighted naive Bayes, the experimental results validated that it indeed improved the prediction accuracy of NB. Schidler and Szeider [15] proposed the SAT-based decision tree method by combining heuristic and exact methods in a novel way, which successfully decreased the depth of the initial decision tree in almost all cases. Ma et al. [16–21] adopted many well-established metaheuristics and the most recent metaheuristics to tune the hyperparameters of SVR and evaluated through nonparametric Friedman and post hoc Nemenyi tests to identify significant differences. Atalla et al. [22] utilized machine learning and graphic analysis to design an automated intelligent recommendation system for academic consulting based on course analysis and performance modeling.

Compared with probability statistical methods, ML-based prediction methods can use the data to mine the law of the change of performance and predict the trend of future performance changes. These algorithms do not require the participation of professionals, but only extract the model from the relevant data. Naive Bayes typically exhibits excellent performance on small-scale datasets, however, it can be highly susceptible to the representation of input data. DT can be applied to samples lacking attribute values and has strong robustness to outliers, however, it is susceptible to overfitting. Artificial neural network performs well on nonlinear data, but the training time is long. The computational complexity of the artificial neural network is directly proportional to the network complexity. SVR has strong generalization ability and can be applied to high-dimensional nonlinear data, but it is sensitive to the selection of parameters and kernel functions. In addition, the time and space complexity of common ML models [23] are shown in Table 1. Where *n* is the number of the training set, *m* is the dimensions of the sample, *c* is the number of categories of Naive Bayes, *p* is the number of nodes in the tree, *n*_*i*_ is the number of neurons in ith layer, *d* is the maximum depth of the decision tree, *t* is the training times and *p* is the number of interneurons, *n*_*SV*_ is the number of the support vectors in SVR.

**Table 1:** The time and space complexity of common ML models.

Model	Time complexity	Space complexity
Logistic Regression	*O*(*n* * *m*)	*O*(*m*)
Naive Bayes	*O*(n * m * c)	*O*(m * c)
Decision Tree	*O*(*n* * log(*n*) * *d*)	*O*(*p*)
Artificial neural networks	*O*(*t* ∑(*n*_1_ *n*_2_ + *n*_2_ *n*_3_ + ⋯)	*O*(*t* * ∑(*n*_1_ *n*_2_ + *n*_2_) + (*n*_2_ *n*_3_ + *n*_3_) + ⋯)
Support Vector Regression	*O*(*n*^2^)	*O*(*n*_*SV*_)

Recently, more and more scholars have been using ML models to predict students’ performance. Baradwaj et al. [24] used DT to evaluate student’s performance in end semester examination. Amrieh et al. [25] established a student’s performance prediction model based on data mining techniques with student’s behavioral features. Saa [26] established a qualitative model to classify and predict the students’ performance based on related personal and social factors. Hooshyar et al. [27] used student’s assignment submission behavior to predict the performance of students with learning difficulties through procrastination behavior. Okubo et al. [28] proposed a method for predicting final grades of students by a recurrent neural network from the log data stored in the educational systems. Sultana et al. [29] used deep neural net to predict the students’ performance. Xie [30] proposed an Attention-based Multi-layer LSTM model to predict student performance based on student demographic data and clickstream data. Hassan et al. [31] and Aljohani et al. [32] used deep long short-term memory model to predict the students’ performance based on clickstream data in the virtual learning environment. The aforementioned references on prediction on student’s performance are summarized in Table 2.

**Table 2:** Students’ performance research in recent years.

Publications	Problems	Methods
Elbadrawy et al. [7]	Student’s performance prediction	Collaborative multi-regression models
Sravani and Bala [8]	Student’s performance prediction	Linear regression model
Baradwaj et al. [24]	Student’s performance evaluation	Decision tree
Amrieh et al. [25]	Student’s performance mining	Bagging, Boosting and Random Forest
Saa [26]	Student’s performance classification and prediction	Decision tree
Hooshyar et al. [27]	Online learning student’s performance prediction	Linear support vector machine
Okubo et al. [28]	Student’s performance prediction	Recurrent neural network
Sultana et al. [29]	Student’s performance prediction	Deep neural net
Xie [30]	Online learning student’s performance prediction	Long-Short Term Memory
Hassan et al. [31]	Student’s performance prediction	Deep Long-Short Term Memory
Aljohani et al. [32]	Student’s performance prediction	Deep Long-Short Term Memory

Different ML models have different characteristics. Different models also have different sensitivity to features. Individual models often reinforce some features and ignore others. Therefore, a single model is difficult to meet the requirements of high precision prediction. The research on combination prediction method has begun to rise. Kotsiantis et al. [33] combined an incremental version of Naive Bayes, the 1-NN and the WINNOW algorithms based on the voting methodology to predict students’ performance. Han et al. [34] established the combination prediction model to predict the students’ performance based on AdaBoost method. Hassan et al. [35] integrated multiple classifiers including DT, artificial neural network and support vector machine to establish a students’ performance classification model.

The main advantage of the combination prediction method is that it can comprehensively utilize the prediction results of multiple predictors and avoid the possible one-sidedness when using a single predictor. The core of the combined prediction method is the calculation of the weight of each predictor. Despite the numerous proposed weight assignment methods, finding the suitable weight configuration remains a challenging task. At present, the most common practice is to assign weights according to the prediction accuracy of the predictor [36,37]. However, when the prediction accuracy gap between the predictors is too large, this method cannot guarantee the integrated results better than the results of a single predictor. Meta-heuristic methods can be used as a solution to find optimized configurations. Genetic algorithms (GA), Particle Swarm Optimization(PSO) and ACO are some popular approaches on which current researches are going on. However, GA and PSO have the disadvantages of falling into a local optimum easily in high-dimensional space and have a low convergence rate in the iterative process.

ACO as a new biological evolution simulating method, which has the advantages of parallel computing, positive feedback search, and satisfactory adaptability can be used to avoid this issue [38,39]. Zhang [40] improved the decision tree classification method and ant colony algorithm, and established a data mining model for student employment and entrepreneurship. Ye et al. [41] proposed two innovative wrapper feature selection methods by integrating the ant colony optimization algorithm and hybrid rice optimization. Zhao [42] proposed a value prediction and analysis method of network documents based on ant colony algorithm. Aghelpour et al. [43] coupled adaptive neuro-fuzzy inference system with ant colony optimization algorithm to realize the 1-, 2-, and 3-days ahead forecasting of daily streamflow. Albashish and Aburomman [44] proposed a heterogeneous ensemble classifier configuration based on ant colony optimization for a multiclass intrusion detection problem. The existing research has proved that the ACO can facilitate the weight assignment for combination prediction method, which has great potential in students’ performance prediction [45–47]. Therefore, an ant colony algorithm is proposed to assign the weights of predictors in this paper.

Students’ performance is an important factor for the evaluation of teaching quality in colleges. The prediction and analysis of students’ performance can guide students’ learning in time. Aiming at the low accuracy problem of single model in students’ performance prediction, a combination prediction method is put forward based on ACO. The main contributions of this paper can be summarized as follows: 1) The ACO algorithm is proposed to determine the weights of ML models in the combination prediction method. The combination prediction method based on ACO improves the stability and generalization ability of the ML model. In addition, the combination prediction method does not need to calculate the optimal parameters of the ML model, thereby saving the process of optimizing model parameters. 2) The combination prediction method is applied to the students’ performance prediction. The results show that the proposed method has an outstanding performance in solving the students’ performance prediction problem. This paper can provide new methods for research in the field of student performance.

The rest of the paper is organized as follows. In section 2, DT, SVR and BP neural network are selected to establish three prediction models. In section 3, the combination prediction method is elaborated. In section 4, a real example about academic performance of students is given to illustrate the proposed method. The conclusions are shown in section 5.

## 2. The selection of single prediction model

Students’ performance prediction is the key and difficult point of education departments. There are many factors that affect students’ performance. Learning interest, family factors, teaching environment, and extracurricular learning all determine the changes and trends of students’ performance to varying degrees. Some of the above factors have a certain correlation and regularity on the impact of students’ performance. For example, there is a positive correlation between learning interest and students’ performance. However, there are many uncertainties and randomness in students’ performance prediction. Therefore, the students’ performance is complex and difficult. Selecting a variety of students’ performance prediction models and combinatorial optimization can improve the prediction effect.

The existing prediction models have differences in the modeling mechanism and the data used for modeling. DT is easy to understand and implement because of its unique tree structure. It is the most used method in the research of students’ performance prediction [48]. The SVR model based on statistical learning theory can approach the nonlinear function with arbitrary precision by mapping the input vector to a high-dimensional space, which is suitable for studying the prediction on students’ performance with complex multi-factor variables. BP neural network can automatically extract reasonable rules between input and output data by learning, and has high accuracy in student performance prediction. In addition, the above three models have great differences in modeling mechanism, which will maximize the prediction effect after integration. After the above analysis, we select DT, SVR and BP neural network to establish single prediction model respectively.

### 2.1 Decision tree

Decision tree algorithm is a widely used machine learning algorithm, which is based on the concept of decision tree and used to solve classification and regression problems. It is a supervised learning algorithm and often used for classification tasks. The algorithm divides data into different classes by constructing a decision tree based on the sample data. It starts from the root node and recursively divides the data into different branches based on the splitting criteria until the leaf node is reached or no more splitting is possible. Each node in the decision tree represents a feature attribute and each edge represents a decision condition. The splitting criteria are determined by the impurity criterion, such as information entropy, Gini index, etc. The decision tree algorithm has the advantages of simple concept, easy to understand and visualize, and can handle various types of data. However, it also has the disadvantages of easy to overfit and sensitive to changes in data. It is usually used in combination with other algorithms to improve performance.

At present, the popular DT algorithms principally include ID3, C4.5 and classification and regression tree (CART). ID3 cannot handle the continuous data. C4.5 makes up for the above problem of ID3. However, when dealing with continuous data, the necessity to sort the information may potentially compromise the prediction performance. The CART algorithm can not only deal with the continuous data, but also suitable for modeling complex data with multiple variables. It has simple rule extraction, high accuracy and strong interpretability.

In this paper, we build a prediction model based on the CART module in sklearn in Python. The setting of important parameters in CART algorithm is shown in Table 3.

**Table 3:** The setting of important parameters in CART algorithm.

Parameters	Value
Maximum depth	5
Judging criterion	gini

### 2.2 Support vector regression

SVR is a machine learning algorithm used for regression analysis. It is a type of kernel-based method that belongs to the family of Support Vector Machines (SVMs). SVR is commonly used for predicting continuous outcomes, such as the target variable in a regression problem. SVR is based on the concept of finding a hyperplane that optimally separates the data, known as the optimal separating hyperplane (OSH). In SVR, the OSH is determined by using support vectors, which are the data points that lie closest to the hyperplane and define its boundary. The algorithm then constructs a model that predicts the target variable based on the features of the input data using the support vectors. SVR has several advantages over traditional regression methods, such as its ability to handle high-dimensional data, small sample sizes, and complex decision boundaries. It also provides a trade-off between model complexity and generalization performance through a regularization parameter, which controls the amount of influence of the support vectors on the model.

In this paper, we build a prediction model based on the SVR module in sklearn in Python. Taking the mean square error as the objective criterion, the k-fold cross validation is used to select the kernel function and allowable error *ε*, penalty parameter *C* and kernel width *γ* dynamically. The search range of each parameter involved in cross-validation is shown in Table 4.

**Table 4:** List of preset parameters in SVR.

Parameters	Value
Kernel function	[Linear, Poly, Sigmoid, RBF]
Allowable error ε	[0.01,0.05,0.10,0.50,1.00]
Penalty parameter C	[10,40,80,120,200,500]
kernel width *γ*	[0.01,0.05,0.10,0.50,1.00]

### 2.3 BP neural network

**Fig 1:**
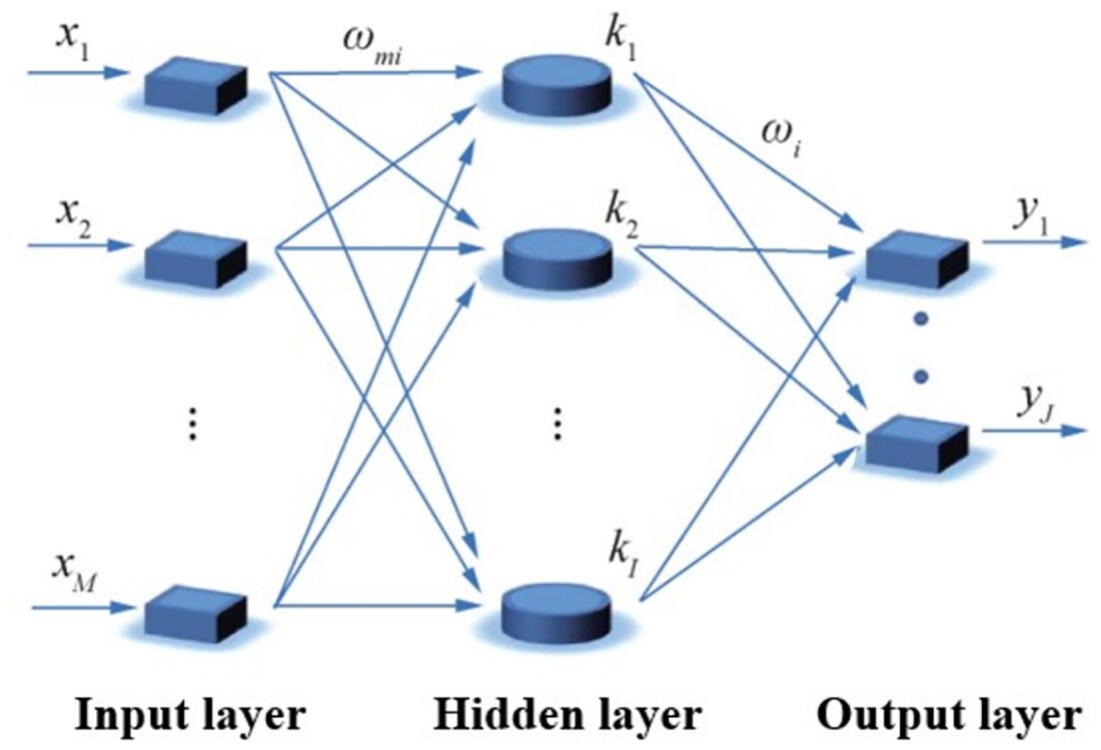
BP neural network structure.

BP neural network is one of the most commonly used neural networks. It is a feedforward neural network with multi-layer structure. In the learning process of BP neural network, the error back propagation method is used. BP neural network generally includes an input layer, an output layer and several hidden layers. Fig 1 shows a simple three-layer network structure with a single hidden layer. Interneurons are the fundamental units in BP neural network that receive multiple input signals. Each input signal has a corresponding weight, which is determined based on the importance of the input to the current neuron. Interneurons weight the input signal and add it to other input signals. After mapping through activation functions, output signals are generated and transmitted to other interneurons. Sigmoid function or linear function with differentiability is often used as activation function. The sample data is propagated forward, that is, from the input layer to the output layer through the hidden layer. When training the weights, the opposite is true. The sample data passes through the hidden layer from the output layer to the input layer. The weights of connections between neurons are corrected along the direction of reducing errors.

In this paper, we build a prediction model based on the BP neural network module in sklearn in Python. The setting of important parameters in BP neural network is shown in Table 5.

**Table 5:** The setting of important parameters in BP neural network.

Parameters	Value
Transfer function	Sigmoid
Hidden layer	5
Epochs	100

## 3. Combination prediction method

Combination prediction model integrates multiple models in a certain way. It can combine the advantages of each single prediction model to better predict the data. The determination of weight is the key to establish the combination prediction model. The predictive performance of the combination prediction model is different with different weights. In this section, we propose an ant colony algorithm to calculate the weight of the combination prediction model.

### 3.1 Combination prediction frame

**Fig 2:**
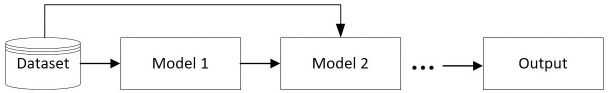
The framework of the serial structure combination prediction model.

The combination prediction model can be divided into two types: serial structure combination prediction model and parallel structure combination prediction model. In the serial structure combination prediction model, the output of the former model is the input of the latter model. Each single model learns sequentially. Fig 2 shows the framework of the serial structure combination prediction model. The performance and the ranking order of the single models have a great influence on the effect of the serial structure combination prediction model. A common practice is that models with high prediction accuracy and difficulty in interpreting should be ranked first.

**Fig 3:**
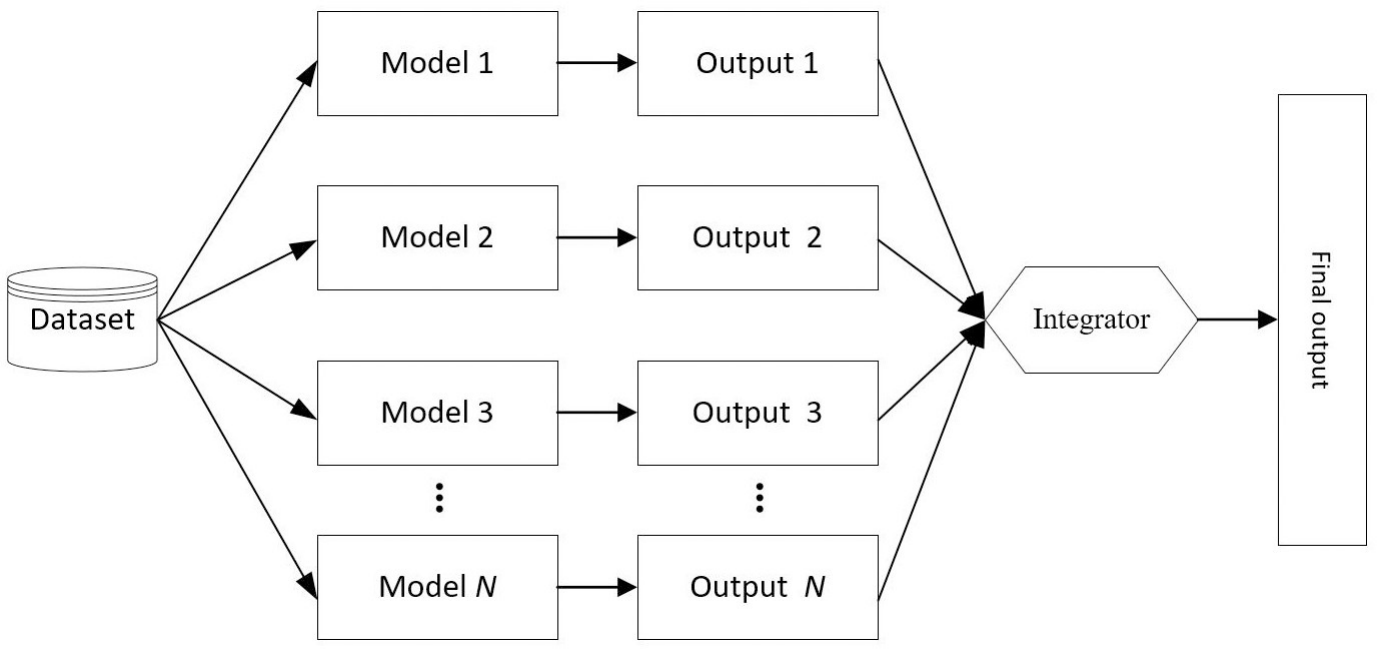
The framework of the parallel structure combination prediction model.

In the parallel structure combination prediction model, the outputs of multiple models are combined through a certain strategy to obtain the final prediction result. Fig 3 shows the framework of the parallel structure combination prediction model. In the process of model building, each model is independent. Specifically, each single model learns simultaneously, and the output of each model is fed into the integrator. Generally, the weighted voting method is used to integrate the output results.

In the serial structure combination prediction model, the error of a single model is transmitted to the subsequently model, which may make the output of the whole combination prediction model unreliable. In addition, the serial structure combination prediction model is not easy to explain. In the parallel structure combination prediction model, the error of a single model will not have a great impact on the combination prediction model. Therefore, it has stronger stability and applicability. Based on the above analysis, we select the parallel structure combination prediction model to predict students’ performance in this paper.

### 3.2 Weight assignation based on ant colony algorithm

**Fig 4:**
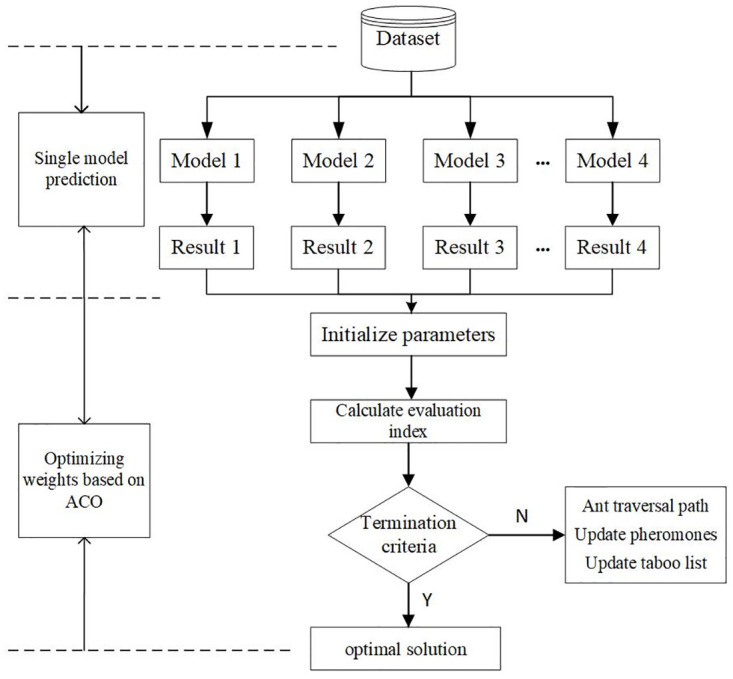
Weight optimization of the combination prediction model based on ant colony algorithm.

Combined prediction method based on weight assignation is actually a process of selecting and utilizing the information of single prediction model. In the literature related to students’ performance prediction, most of them assign weights according to the accuracy of single prediction model. This strategy does not take into account the possible values of the instance under test. The weight of each single prediction model is different when facing different test samples. Therefore, we propose an ant colony algorithm to calculate the weight of the single prediction model in this paper, which realizes the dynamic optimization of the weights. Fig 4 shows the flow chart of the weight optimization of the combination prediction model based on ant colony algorithm.

Ant colony algorithm is a bionic iterative search algorithm that simulates the foraging behavior of ants. The main advantage of ant colony algorithm in solving combinatorial optimization problem is that it has strong self-organization and adaptability. The specific implementation steps of applying ant colony algorithm to determine the weight of each single model of combination prediction model are as follows:

**Step 1.** Weight initialization. First, we set the value range of the weight to be [0,1]. Then, the weight value interval is divided into 100 parts on average, i.e. [0, 0.01], [0.01, 0.02], …,[0.99, 1.00]. Assuming that there are *N* single models involved in the combined prediction method, each value interval can be expressed as [*X*_*ij*_, *X*_(*i*(*j*+1)_], *i* = 1, 2,⋯, *N*, *j* = 0, 1, ⋯, 99. Simultaneously, we initialize the pheromone concentration of each interval to be 1.
**Step 2.** Parameter initialization. There are three parameters that affect the performance of the ACO algorithm. The information heuristic factor *α* affects the probability that a new ant chooses the weight interval passed by the previous ant. The larger the value of *α*, the greater the probability that the new ant will choose the weight interval that the previous ant has passed through. The smaller the value of *α*, the smaller the search range of the ant colony, and the easier it is to fall into the local optimal solution. The expected heuristic factor *β* affects the probability of ant colony selecting the local optimum interval. The larger the value of *β*, the faster the iterative convergence speed, and the easier it is to fall into the local relative optimal solution. The information volatilization factor *ρ* determines the residual pheromone concentration in each interval. The smaller the value of *ρ*, the higher the concentration of residual pheromones in each interval, the larger the search range of the ant colony, and the slower the convergence of the algorithm. The larger the value of *ρ*, the smaller the residual pheromone concentration in each interval, and the algorithm is prone to getting stuck in local optima.
**Step 3.** Ant colony searching. The ant colony searches each weight interval of each single prediction model in turn. The specific search process of a single ant is as follows:
1) Initialize the ants’ birth positions and set the taboo table.
2) The transition probability, that is, the probability that the ants select each weight interval of the single prediction model, is calculated by the following formula:
$$p_{j}^{k}=\left\{\begin{array}{cc} \frac{{\left[\tau_{j}\right]}^{\alpha }∙{\left[n_{j}\right]}^{\beta }}{\sum_{s\in J_{k}}{\left[\tau_{s}\right]}^{\alpha }∙{\left[n_{s}\right]}^{\beta }}, & j\in J_{k}\\ 0, & otherwise \end{array}\right.$$ (1)
  $p_{j}^{k}$ represents the probability that ant *k* moves to the weight interval *j*, *j* = 1, 2, ⋯, 99. *τ*_*j*_ is the pheromone quantity in weight interval *j*. *n*_*j*_ is the information expectation heuristic parameter of weight interval *j*. *J*_*k*_ is the set of weight intervals that ant *k* can select subsequently. *α* is the information heuristic factor and *β* is the expected heuristic factor.
3) Determine the weight interval by roulette. First, we obtain the total probability *P* by summing the selection probability of each weight interval of the prediction model *p*_*j*_, *j* = 1, 2, ⋯, 99. Then a random probability is generated between the [0, *P*] and subtracted from the selection probability of each weight interval in turn. The first weight interval with probability less than 0 is the final weight interval selected by ants.
4) Search the weight interval of other single model prediction model in turn.
**Step 4.** Pheromone update. After each iteration, the pheromone concentration of each weight interval needs to be updated. We select the ant week model based on global information update to calculate and update the pheromone of each weight interval in this paper. After all ants in the ant colony complete a search, the evaluation index of the combined prediction method under the current selected weight interval is calculated. The optimal ant path is obtained according to the evaluation index. The pheromone of the current selected weight interval is updated by
$$\tau^{\left(i\right)}=(1-\rho )\tau^{\left(i-1\right)}+M$$ (2)
  The pheromone of other weight intervals is updated by
$$\tau^{\left(i\right)}=(1-\rho )\tau^{\left(i-1\right)}$$ (3)
  where *τ*_*i*_ is the pheromone increment in the weight interval of the *i*th iteration. *ρ* is the information volatilization factor. *M* is the evaluation index of the combination prediction method under the optimal weight interval.
  In this paper, we take the mean squared error (MSE) as the evaluation index. Let *y*_*i*_ be the observed values and $\hat{y_{i}}$ be the predicted values, then
$$MSE=\frac{1}{n}\sum_{i=1}^{n}{\left(y_{i}-\hat{y_{i}}\right)}^{2}$$ (4)
  where *n* is the number of samples. A smaller MSE value indicates a better fighting capability.

### 3.3 Time complexity of the combination prediction method

The time complexity of the combination prediction method depends on the part of the method with the highest time complexity, which is the time complexity of the ant colony algorithm. The time complexity of ant colony algorithm is shown below.

When *n* is large enough, the influence of lower powers can be ignored. As shown in the table above, the time complexity of the ant colony algorithm is:

$$T\left(n\right)=O(t∙n^{2}∙m)$$

Where *n* is the number of the training set, *m* is the number of the ants, *t* is the maximum iterations.

## 4. Experimental study

In this paper, the mathematical performance data of 240 students in five classes of the second grade in a vocational college in Hefei are selected as the research object. The data of students’ learning score comes from the college’s educational administration system. The data of students’ learning behavior is from the questionnaire of student study behavior habit. Among them, 180 samples are used as the training data and the remaining 60 samples are used as the test data. Each sample contains 18 features, as shown in Table 6. The input features 1–8 in Table 7 are categorical. The numerical value 1–5 of each feature stand for option A-E in the questionnaire of student study behavior habit in S1 Appendix. For example, the numerical value 1–5 in the No.4 feature education level of parents stand for bachelor degree or above, senior high school, junior high school, primary school and uneducated, respectively.

**Table 6:** The features of each student sample.

Category	NO.	Feature	Value
Basic information	1	Sex	{1,2}
	2	Native place	{1,2,3,4,5}
	3	Semester	{1,2}
	4	Education level of parents	{1,2,3,4,5}
	5	Work as a student cadre	{0,1}
Interest	6	Interest in the course	{1,2,3,4}
	7	The degree of keeping up with the class	{1,2,3,4}
	8	Learning initiative	{1,2,3,4}
Behavior in class	9	Number of absenteeism	[0,25]
	10	Frequency of distraction	[5,47]
	11	Average number of hands raised	[0,12]
	12	Number of questions answered	[0,9]
	13	Number of assignments submitted	[0,20]
	14	Number of interactions between teachers and students	[0,17]
	15	Number of group discussions attended	[0,10]
Behavior outside class	16	Time of study this course outside the class	[5,65]
	17	Study extracurricular material time	[0,12]
	18	Online viewing time	[0,60]

**Table 7:** Time complexity of the ant colony algorithm.

NO.	Step	Time complexity
1	Parameter initialization	*O*(*n*^2^ + *m*)
2	Ant Taboo Table Setting	*O*(*m*)
3	Construction of solutions for each ant	*O*(*n*^2^*m*)
4	Evaluation of solutions and trajectory updates	*O*(*n*^2^*m*)
5	Update of pheromone trajectory concentration	*O*(*n*^2^)
6	Determine whether the termination condition of the algorithm has been met (reaching the maximum iterations *t*)	*O*(*mm*)
7	Result output	*O*(1)

### 4.1 Data preparation

To eliminate the influence of the different dimensions on the numerical values, further normalization of data is needed. The normalization formula is as follows.

$$a_{ij}^{'}=\frac{a_{ij}-a_{imin}}{a_{imax}-a_{imin}}$$ (5)

Where *a*_*ij*_ is the initial sample data to be normalized, *a*_*imin*_ and *a*_*imax*_ are the minimum and maximum values in the column sample values.

### 4.2 Experimental study

All experiments are run on Intel Core i5-1035 8 GB, the Microsoft Windows 10 operating system and the development environment of Python 3.6.6, PyCharm 2021.1. During the model training process, *k* fold cross-validation is adopted. The value of *k* in the experiment is set to 10. The parameter settings are shown in Table 8.

**Table 8:** List of preset parameters in the combination prediction method.

Parameters	Value
Information heuristic factor *α*	1.0
Expected heuristic factor *β*	0.5
Information volatilization factor *ρ*	0.5
Population size *m*	100
Iteration time	500

We apply the combination prediction method to predict the students’ performance, and the results are shown in Table 9.

**Table 9:** The results of combination prediction method in students’ performance prediction.

Method	The optimal weight combination	MSE
combination prediction method	[0.09, 0.27, 0.64]	0.0089

**Fig 5:**
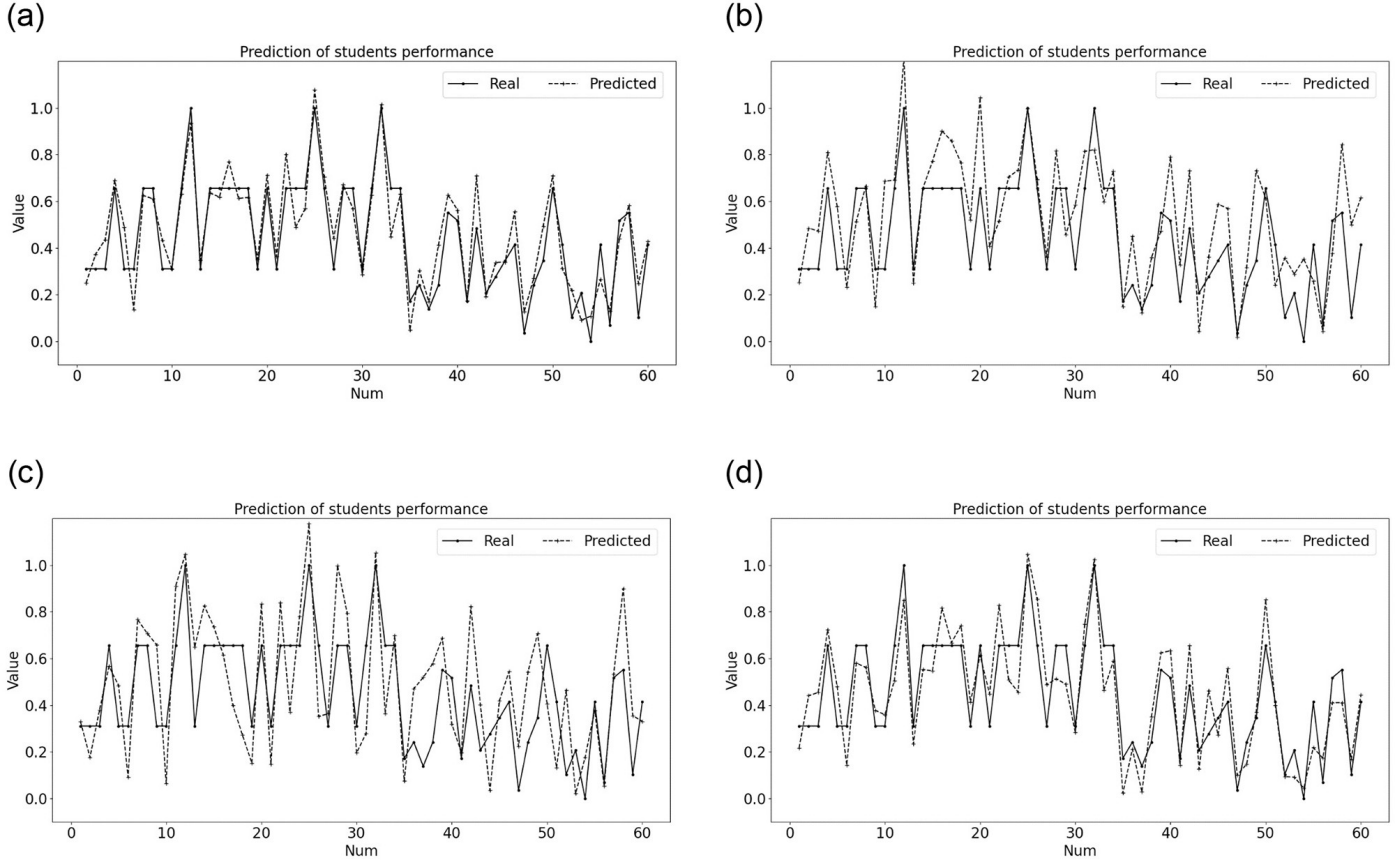
The predicted results of students’ performance based on combination prediction method and three single models.

From Table 7, we can know that the combination prediction method has an outstanding performance on predicting the students’ performance. The optimal weight combination of the single model DT, SVR, BP neural network is [0.09, 0.27, 0.64]. The MSE of the combination prediction method on predicting the students’ performance is 0.0089. The prediction results of students’ performance based on the combination prediction method and three single models are shown in Fig 5.

Fig 5(a) shows the predicted results of combination prediction method. We can see that the predicted value fit the real value well. Fig 5(b)–5(d) respectively show the predicted results of DT model, SVR model and BP neural network. The comparison of performance between combination prediction method and three single models is shown in Table 10. From Fig 5 and Table 10, it can be clearly seen that the predicted result of the combination prediction method is better than that of any single model. However, as shown in Table 10, the combination prediction method has the highest running time, which can be said that it trades time for accuracy. How to reduce the time complexity and running time is a future research direction for us. First, running time may be further reduced by exploring more computational-efficient ML models and faster parameter tuning mechanism. Moreover, parallelization techniques and methods are worth exploring and utilizing to improve learning performance and reduce the computational cost in the model.

**Table 10:** Comparison of performance between combination prediction method and three single models.

Method	MSE	Running time (second)
DT	0.0326	1.24
SVR	0.0229	0.56
BP neural network	0.0148	3.27
Combination prediction method	0.0089	57.52

**Fig 6:**
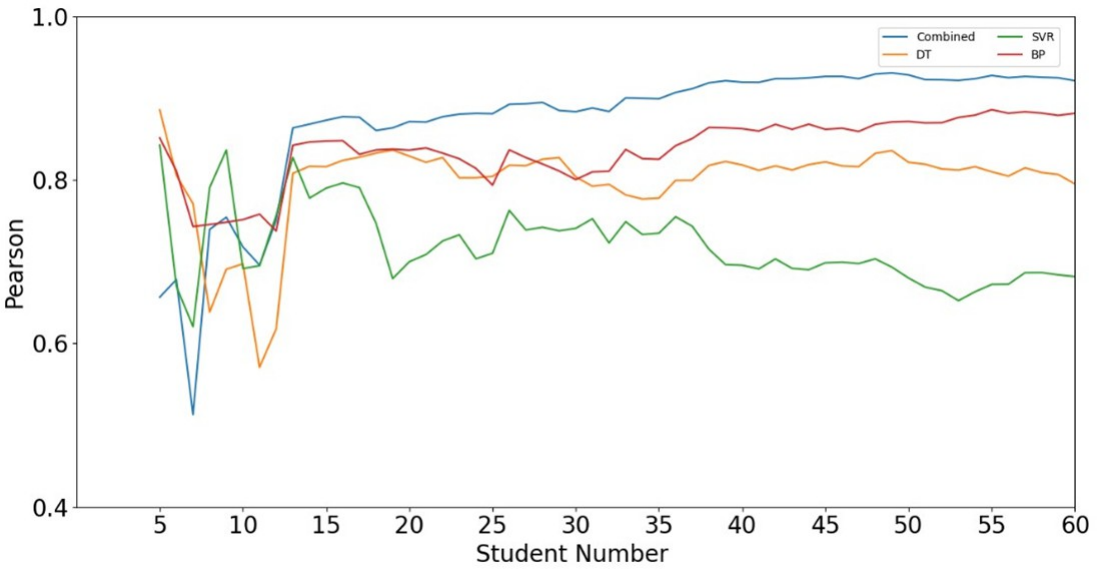
The accumulative Pearson’s correlation coefficient between the real value and the predicted value.

Fig 6 shows the accumulative Pearson’s correlation coefficient between the real value and the predicted value. As can be seen from Fig 6, the correlation calculated by combination prediction method is significantly higher than the other three models. In addition, after number 13, there is a strong correlation between the real value and the predicted value of the combined method with the increase of the number of students. The Pearson’s correlation coefficient calculated by the combined method of the total test data is 0.9206.

**Fig 7:**
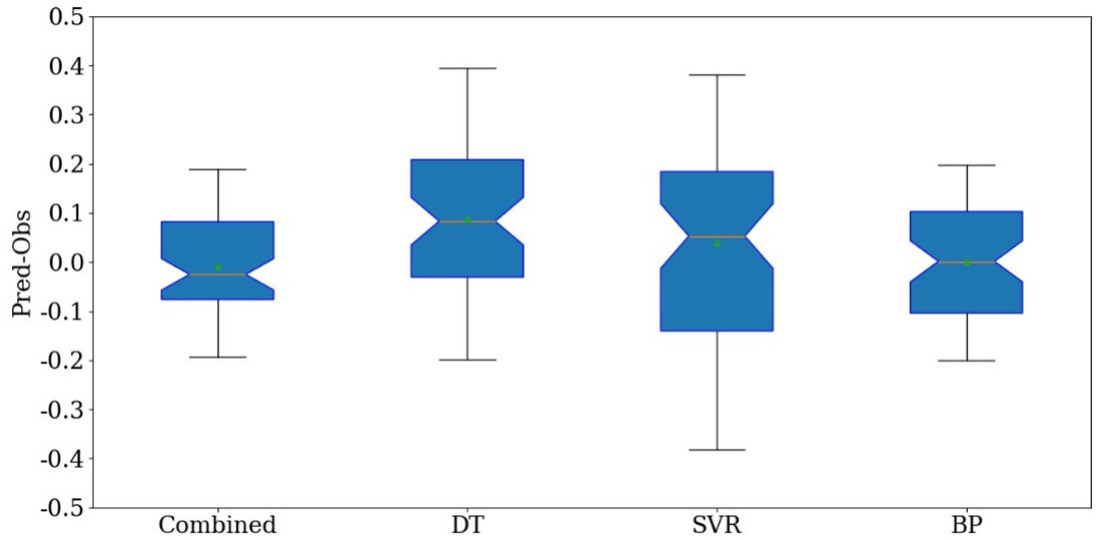
Boxplot representing the difference between the real value and the predicted value.

Fig 7 shows the degree of fluctuation of errors between the real values and predicted value. From Fig 7, we can see that the prediction effect of the combined method is significantly higher than that of the other three models. The errors obtained by the combined method are between -0.2 and 0.2. In addition, there are almost no outliers in the boxplot of the combined method. Comprehensive analysis of Figs 5–7 shows that the combination prediction method has a satisfactory effect on predicting students’ performance.

To verify the superiority of the combination prediction method, other methods random forest (RF), XGBoost, grid search random forest (GS-RF), grid search XGBoost (GS-XGBoost), particle swarm algorithm SVR (PSO-SVR) and improved duel algorithm SVR (IDA-SVR) [49] are selected to compare with the combination prediction method. The population size and iteration times of the PSO-SVR and IDA-SVR are set the same as those of ACO algorithm. Table 11 shows the results of the combination prediction method and the comparative methods on the students’ performance prediction problem. We find that the prediction accuracy of the combination prediction method is better than that of the selected comparative methods. In addition, as shown in Table 11, the combination prediction method has the lowest running time compared with other methods based on intelligent optimization algorithm (PSO-SVR and IDA-SVR). This is because the first two methods involve optimizing parameters, and each iteration requires retraining the model. The proposed combination prediction method only needs to optimize the weights of three single models, and does not need to repeat the training model.

**Table 11:** Comparison of performance between combination prediction method and other methods.

Method	MSE	Running time (second)
RF	0.0175	3.74
XGBoost	0.0173	7.43
GS-RF	0.0156	365.22
GS-XGBoost	0.0131	443.52
PSO-SVR	0.0117	90.58
IDA-SVR	0.0092	105.17
Combination prediction method	0.0089	57.52

## 5. Conclusions

In this paper, a combination prediction method of students’ performance is proposed based on ant colony algorithm. Considering the characteristics of students’ learning behavior and the characteristics of the models, DT, SVR and BP neural network are selected to establish three prediction models. The ant colony algorithm is designed to calculate the weight of each model of the combined prediction method. The experimental results show that the combination prediction method has excellent performance in solving the prediction problem of students’ performance.

Although the proposed method performs well among many methods, it still has some limitations. First, the ACO algorithm has some instability. For example, the initial values of the parameters to be optimized are given randomly, and different initial values will have different effects on the results. In addition, even though the ACO algorithm provides the possibility of global search, it can not ensure that it converges to the global best. Second, the combination prediction method can get much better results than other algorithms on small sample training set. However, when the sample dimension is large, the time complexity of the single model will increase, which will greatly reduce the efficiency of predictor. Third, the ACO algorithm optimizes the weights by training individuals on the training set and evaluating the scores on the testing set. The more iterations of optimization, the higher the accuracy. In other words, the proposed model trades time for accuracy to a large extent.

To solve the above limitations, our study can be extended in the following future research directions. With the development of computer technology, the number of layers of neural networks that can handle is increasing, and the performance of deep learning methods has surpassed machine learning in many fields. In addition, to improve the performance of prediction model, it is necessary to improve the objective function and constraint conditions of the prediction model based on the problem itself.

## Supporting information

S1 Dataset The data set of student study behavior habit in this study for training. (TXT)

S1 Appendix (DOCX)
