# Supplementary material for: Combination prediction method of students’ performance based on ant colony algorithm
Source: PLoS One. 2024 Mar 11;19(3):e0300010. doi: 10.1371/journal.pone.0300010 (PMC10927126; doi:10.1371/journal.pone.0300010)
Supplement: S1 Appendix — (DOCX) [file pone.0300010.s002.docx]

**Appendix**

**The acronyms in the paper**

| **Acronym** | **Definition** |
| --- | --- |
| ML | Machine learning |
| DT | Decision tree |
| SVR | Support vector regression |
| SVM | Support vector machine |
| BP | BP neural network |
| ACO | Ant colony algorithm |
| CART | Classification and regression tree |
| MSE | Mean squared error |
| PSO | Particle swarm algorithm |
| IDA | Improved duel algorithm |
| RF | Random forest |
| GS | Grid search |

**Student study behavior habit survey questionnaire**

Dear students,

Hello! We are conducting a questionnaire survey on the learning behaviors and habits of students in our school. Please spare a few minutes to help complete our questionnaire. Thank you very much！

Academy： Major： Year： Gender：

**Part 1 Basic information**

1. Your gender（ ）

A. Male

B. Female

1. Your native place（ ）

A. Municipality directly under the central Government

B. City

C. County

D. Town

E. Village

1. Your semester（ ）

A. First

B. Second

1. Education level of parents（ ）

A. Bachelor degree or above

B. Senior high school

C. Junior high school

D. Primary school

E. Uneducated

1. Work as a student cadre（ ）

A. Yes

B. No

**Part 2 Interest**

1. Your interest in the course（ ）

A. Very

B. In general

C. Very few

D. No

1. The degree of keeping up with the class（ ）

A. Very

B. In general

C. Very few

D. No

1. Learning initiative（ ）

A. Very

B. In general

C. Very few

D. No

**Part 3 Behavior in class**

1. Number of absenteeism in this semester
2. Frequency of distraction in this semester
3. Average number of hands raised in this semester
4. Number of questions answered in this semester
5. Number of assignments submitted in this semester
6. Number of interactions between teachers and students in this semester
7. Number of group discussions attended in this semester

**Part 4 Behavior outside class**

1. Time of study this course outside the class in this semester
2. Study extracurricular material time in this semester
3. Online viewing time in this semester

***Note:*** *First, this study did not involve human clinical trials or animal experiments, but only a questionnaire survey of students' learning status. Therefore, there is no unethical behavior in this study. Second, this study was conducted in a typical teaching scenario with low risk. Then, the questionnaire survey adopts the principle of voluntary filling by students. If the surveyed person agrees to fill out the questionnaire, they can submit it without signing. If he does not agree to accept the survey, he will refuse to fill out the questionnaire. Finally, the participants in the questionnaire survey are college students from Hefei Preschool Education College, all of whom are adults. Based on the above considerations, according to relevant institutional guidelines and national laws and regulations, this study does not require ethical approval.*

**Description on a survey questionnaire for students’ study behavior habits**

**1 Background**

Through the investigation of students' learning behavior, we explore the common problems of students' learning attitude and learning behavior habits. According to the conclusion of the questionnaire survey, it provides realistic basis for the project research, and explores the countermeasures to solve the problem.

**2 Purpose**

The quality of students' learning behavior habits directly affects their academic performance and the formation of students' personality and personality. Positive learning habits are the basic guarantee for students to learn smoothly. According to the usual observation and understanding, we found that some students have not developed a positive learning attitude and positive learning habits. As a result, students lack the ability and motivation to continue learning. In order to find the effective ways and methods to cultivate students' learning habits, the study attitude and study habits of students in our school are investigated and studied.

**3 Investigation institution**

Department of Public Teaching, Hefei Preschool Education College, Anhui Hefei 230011, China

**4 Respondent**

The student of Hefei Preschool Education College

**5 Investigation method**

This investigation was conducted by visiting classes and dormitories in the form of questionnaires. The questionnaire is principally designed and investigated from four aspects, including basic information, interest, behavior in class, behavior outside class.

**6 Investigation time**

September 8, 2021
